# miRNA nanoencapsulation to regulate the programming of the blood-brain barrier permeability by hypoxia

**DOI:** 10.1016/j.crphar.2022.100129

**Published:** 2022-09-15

**Authors:** Esteban G. Figueroa, Aitor Caballero-Román, Josep R. Ticó, Montserrat Miñarro, Anna Nardi-Ricart, Alejandro González-Candia

**Affiliations:** a Laboratory of Fetal Neuroprogramming, Institute of Health Sciences, Universidad de O'Higgins, Rancagua, Chile; b Pharmacy and Pharmaceutical Technology, and Physical Chemistry Department, Faculty of Pharmacy and Food Sciences, University of Barcelona, Avinguda Joan XXIII, 27-31, 08028, Barcelona, Spain

**Keywords:** miRNA, Epigenetic, Hypoxia, Nanoparticle, Nose-to-brain

## Abstract

Central nervous system (CNS)-related diseases are difficult to treat as most therapeutic agents they cannot reach the brain tissue, mainly due to the blood-brain barrier (BBB), arguably the tightest barrier between the human body and cerebral parenchyma, which routinely excludes most xenobiotic therapeutics compounds. The BBB is a multicellular complex that structurally forms the neurovascular unit (NVU) and is organized by neuro-endothelial and glial cells. BBB breakdown and dysfunction from the cerebrovascular cells lead to leakages of systemic components from the blood into the CNS, contributing to neurological deficits. Understanding the molecular mechanisms that regulate BBB permeability and disruption is essential for establishing future therapeutic strategies to restore permeability and improve cerebrovascular health. MicroRNAs (miRNAs), a type of small non-coding RNAs, are emerging as an important regulator of BBB integrity by modulating gene expression by targeting mRNA transcripts. miRNAs is implicated in the development and progression of various illnesses. Conversely, nanoparticle carriers offer unprecedented opportunities for cell-specific controlled delivery of miRNAs for therapeutic purposes. In this sense, we present in this graphical review critical evidence in the regulation of cell junction expression mediated by miRNAs induced by hypoxia and for the use of nanoparticles for the delivery of miRNA-based therapeutics in the treatment of BBB permeability.

## 1 Introduction

The microvascular system, responsible for delivering the oxygen and nutrients into the brain parenchyma of the central nervous system (CNS), possess unique properties that differentiate it from the peripheral vascular system. This complex, known as the blood-brain barrier (BBB), is an intricate physiological barrier that separates the blood network from the neuronal-glial cells from the CNS (Gonzalez-Candia et al., 2021; Heinbockel and Zhou, 2021). The BBB is responsible for the maintenance of selective transport to the brain and its protection, ensuring correct cellular homeostasis in the organ; thanks to its specialized multicellular structure: the neurovascular unit (NVU) (Schaffenrath and Keller, 2020). NVU is constituted by different types of cells such as pericytes, astrocytes, microglia, neurons, and endothelial cells. This unit has more protein expression than forming cellular junctions, significantly limiting the paracellular flux of solute and allowing low rates of transcytosis compared to peripheral vascular cells. Peripheral vascular cells substantially restrict the vesicle-mediated transcellular movement of solutes compared to the endothelial cell junctions observed at other sites in the vascular territory (McConnell and Mishra, 2022). This feature, which depends mainly on the different interconnections between cells through a differential expression of tight junctions (TJ), adherents junctions (AJ), and gap junctions (GJ) in the inter-endothelial cleft, provides high integrity and selective permeability to the BBB (Bai et al., 2022). This restrictive nature of the BBB provides an obstacle for drug delivery to the CNS; thus, significant efforts have been made to generate methods to modulate or bypass the BBB for the delivery of therapeutics (Pandit et al., 2020). However, the loss of TJs is associated with a breakdown of the BBB with an increase in neurodegenerative disorders and acute CNS-associated diseases. Loss of some of these barrier properties during neurological conditions, including stroke, brain traumas, and neurodegenerative disorders, is a major factor in the progression of these diseases (Zlokovic, 2008; O’Leary and Campbell, 2021).

Recent genome-wide studies have revealed that eukaryotic genomes are extensively transcribed to produce thousands of non-coding RNAs (ncRNAs). Several ncRNAs are directly involved in protein translation, such as messenger RNAs (mRNAs), transfer RNAs (tRNAs), and ribosomal RNAs (rRNAs). Nonetheless, a ncRNA can be broadly classified into small ncRNAs (less than 200 nucleotides), sequences that lately have not only been identified as critical regulators of gene expression, but also as assistants in multiple biological and physiological processes (Lee et al., 2019). These sequences, known as microRNA (miRNAs), are small, endogenous, single-stranded ncRNA molecules that are typically 19–25 nucleotides long and are involved in transcriptional and post-transcriptional gene expression regulation by affecting the translation as well as stability of the mRNAs (Yang et al., 2021). Lately, miRNAs have been proposed as pharmacological molecules for several pathologies, as they can act by means of miRNA inhibition therapy, miRNA replacement, or induction therapy (Lee et al., 2019). The most common therapeutic strategy consists of generating a single-stranded oligonucleotide with a complementary sequence to a mature miRNA (identified as the drug target) or restoring intracellular concentrations of a miRNA (miRNA mimics) introduced into cells to provide an exogenous source of additional miRNAs restoring physiological function (Yang et al., 2021).

Understanding the main advantages and disadvantages of the different strategies to overcome the challenges of an efficient miRNA delivery, contributes to developing safe and effective therapeutic approaches to maintain BBB integrity in many CNS diseases. Despite the progressively increasing knowledge of miRNA ability to mediate biological processes, critical hurdles related to miRNA delivery should be overcome to promote their translational application. The administration of miRNAs without a vehicle (naked miRNAs) is not recommended owing to a poor uptake by cells due to their electrostatic properties (Scomparin et al., 2015), along with undesired physiological effects, short half-life in systemic circulation (Mori et al., 2019), and limited stability in the bloodstream because of their rapid degradation or inactivation by nucleases (Lee et al., 2019). Given these points, in this graphical abstract, we provide an overview of miRNAs and their regulation of BBB permeability generated by epigenetic modifications induced by hypoxia. Subsequently, the preparation, characterization, and application of nanoparticles (NPs) for delivering miRNA-based agents is discussed.

## 2 Hypoxia and epigenetic programming of BBB

**Fig. 1:**
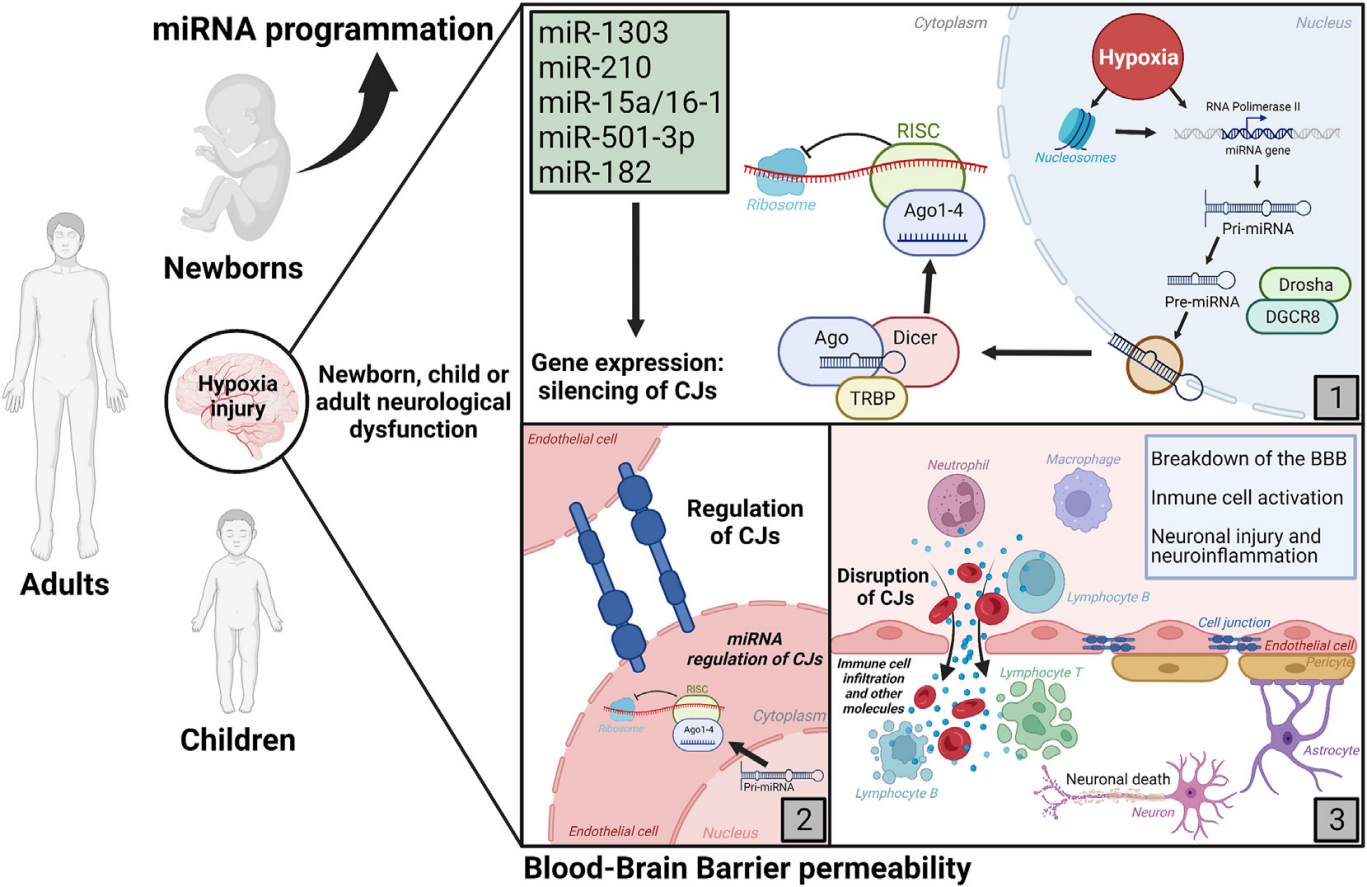
Programming by hypoxia and altered BBB permeability. During fetal development and young-adult life, being subjected to hypoxia can activate epigenetic mechanisms such as miRNAs. (1) Hypoxia can alter gene expression by histone methylations and activation of miRNA expression, RNA polymerase II forms a pri-miRNA and then binds to Drosha, a ribonuclease 3, and DGCR8 (DiGeorge critical region 8), enzymes that catalyze a reaction eliminating the miRNA stem obtaining a precursor called pre-miRNA. The pre-miRNA is exported into the cytoplasm through exportin-5 (EXP5), then an endoribonucleolytic reaction catalyzed by Dicer and transactivating response RNA-binding protein (TRBP) generates a mature double-stranded miRNA. The miRNA is loaded into an RNA-induced silencing complex (RISC), which together with argonaute (AGO), degrades double-stranded RNA, releasing the single-stranded miRNA to silence the target mRNA then. (2) The miRNAs: miR-1303, miR-210, miR15a/16–1, mi-R501–3p and miR-182, expressed under pathological conditions such as hypoxia, alter the expression of protein junctions. (3) Alteration in the expression of protein junctions results in increased BBB permeability, which translates into the BBB becoming permeable to different molecules found in the bloodstream, causing inflammation and neuronal death.

The molecular mechanisms of hypoxia response are complex, even affecting gene expression. The main involved component is the hypoxia-inducible factor (HIF), which promotes cellular processes in response to hypoxia. Changes in gene expression can be altered by HIF, which can even program our early intrauterine and late-life (Herrera and González-Candia, 2021) or during postnatal life (Semenza 2011). However, cellular programming generated by epigenetic mechanisms can alter the expression pattern of a given gene without altering its DNA sequence (Radford, 2018). Epigenetic mechanisms include DNA methylation/demethylation, post-translational changes in histones, and non-coding RNAs such as miRNAs. miRNAs are produced using RNA polymerase II transcription, forming a primary miRNA (pri-miRNA), which is processed in the nucleus into a smaller precursor (pre-miRNA). The pre-miRNA is exported to the cytoplasm to be processed by the ribonuclease DICER, producing a miRNA duplex that would be processed by the RNA-induced silencing complex (RISC) and argonaute proteins (AGO1-4) to obtain a single-stranded miRNA. At this point, RISC silences protein expression by mRNA degradation (Lee et al., 2019) (Fig. 1). miRNAs can be found in different biological fluids such as plasma, cerebrospinal fluid, saliva, urine, milk, and others (Godoy et al., 2018). These extracellular miRNAs travel in vesicles and exosomes bound to AGO proteins to avoid the loss of their stability. This fact implies that hypoxia can produce alterations in different cell types that target BBB regulation (Zhang et al., 2015).

In recent years, there has been increasing evidence of miRNA regulation of cellular junctions in different pathologies. For example, in an *in vitro* BBB model made of endothelial cells, miR-1303 regulates BBB permeability due to the increased expression of Matrix metallopeptidase 9 (MMP-9) and the deregulated integrity of endothelial cells of the NVU (Song et al., 2018). Inhibition of miR-210 has been shown to decrease permeability by increasing occludin and β-catenin (Ma et al., 2017). In addition, miR-15a/16–1 induced by hypoxia in a murine stroke model, affects claudin-5 expression in endothelial cells, increasing permeability (Ma et al., 2020). In contrast, in vascular cognitive impairment, a high expression of inflammatory mediators has been associated with increased intracellular concentrations of miR-501–3p, a factor that is able to decrease the expression of Zonula occludens-1 (ZO-1) by complementary bases. In addition, when miR-501–3p was inhibited with an anti-miRNA (antagomiR), ZO-1 expression recovered, normalizing BBB permeability (Toyama et al., 2018). Studies of miR-182 have shown that it downregulated claudin-5 expression in brain endothelial cells, increasing BBB permeability (Wang et al., 2021; Chakraborty et al., 2020).

In this sense, an increasingly number of miRNAs are related to BBB permeability, specifically by alteration of TJs. For this reason, we are studying ways to inhibit their expression through antagomiRs, nucleotide sequences complementary to the miRNA targets, allowing the formation of a double-stranded RNA and its subsequent degradation (Cerro-Herreros et al., 2020; Gambari et al., 2016; Hanna et al., 2019). However, it is necessary to study pharmaceutical drug delivery systems that allow the correct loading of antagomiRs to reach the target.

## 3 NPs and strategies to cross the BBB

The study and development of nanostructures is one of the research fields with more potential in pharmaceutical technology and galenic pharmacy. Nanosystems act as carriers to deliver drugs in certain cells or target tissues, allowing a higher control of molecules with therapeutic activity. Nanocomplexes or nanosystems are biocompatible structures whose size range between 1 and 100 ​nm. Due to these characteristics, several studies are focused on the improvement of nanostructures in the field of pharmaceutic nanotechnology, with the aim of ameliorate their drug release profiles and their ability to cross challenging barriers like the BBB (Teleanu et al., 2018).

**Fig. 2:**
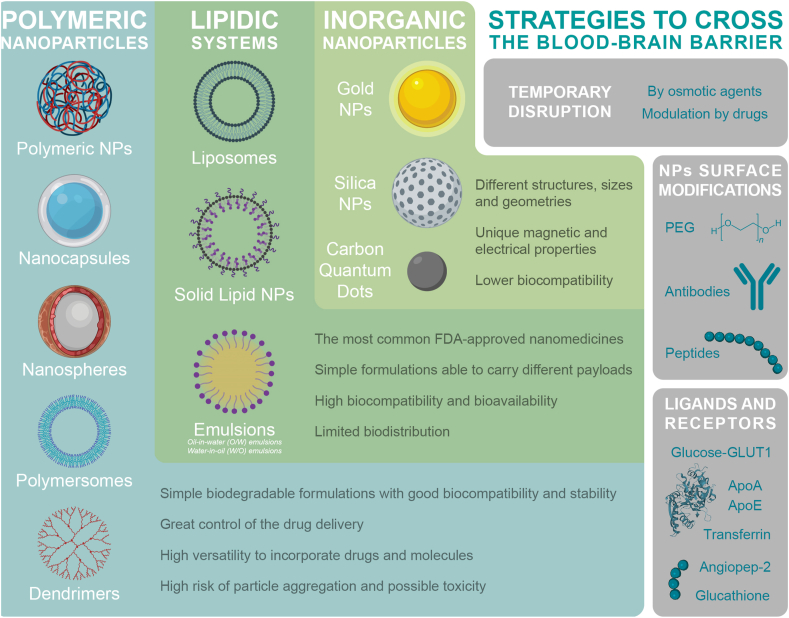
Types of nanoparticles (NPs) and strategies to cross the blood-brain barrier. *FDA (Food and Drug Administration); PEG (polyethylene glycol); GLUT-1 (Glucose transporter-1); ApoA (apolipoprotein A); ApoE (apolipoprotein E).*

As previously stated, one of the factors involved in the low permeability of the BBB is the presence of intercellular cell junction complexes (Ceña and Játiva, 2018). To enhance the delivery of molecules to the brain, several strategies to cross the BBB can be used (Fig. 2): chemical modifications of administered drug, temporary disruption of protein junctions, and the use and functionalization of nanoparticles (NPs) targeting the brain; administered to the brain by surgery or by the use of different vehicles and devices (Zhou et al., 2018; Li et al., 2021).

Usually, the first approach to cross the BBB is the chemical modification of the drug itself by variating its polarity or by conjugating it with other molecules. This strategy can be combined, as mentioned above, with temporary disruption of protein junctions to enhance the permeability of the BBB and to improve the entrance of selected APIs. Temporary disruption can be achieved by using drugs, osmotic agents, or ultrasounds. However, these are risky procedures and techniques since the hemostatic function of the BBB can be compromised; several compounds and molecules that are not supposed to cross the barrier could penetrate.

Nonetheless, the development of NPs is one of the most used strategies to achieve a good drug delivery in the brain. These non-toxic systems, formulated with biocompatible materials in the nanometric scale, usually have a long residence time and positive charge (Teleanu et al., 2018). Additionally, NPs can be improved for medical purposes by refining their ability to carry hydrophilic or hydrophobic APIs and enhancing their chemical and biological stability. All the above-mentioned modifications make NPs a suitable drug delivery system that can be administered in a wide variety of routes, including parenteral, inhalatory and nasal (Sosnik et al., 2014).

As seen in Fig. 2, NPs that are able to cross the BBB can be classified by their composition:

- Polymeric NPs

Formed by natural or synthetic materials, ranging from monomers to polymers with different compositions and structures. This kind of NPs can be prepared, among other techniques, by emulsification, spray drying and nanoprecipitation. Their morphology and characteristics depend on the used materials and technique, achieving NPs with a polymeric nucleus, nanocapsules, nanospheres, polymersomes or dendrimers (Mitchell et al., 2021). Polymeric NPs have several advantages as their versatility to have their surface modified and their ability to incorporate hydrophilic or hydrophobic drugs with different molecular weights like proteins, vaccines or miRNAs.

- Lipidic systems

Lipidic systems are characterized by having at least a lipidic layer around one or more intern aqueous compartments. They can be prepared by different methods, obtaining liposomes, solid lipid NPs (LNPs) or emulsions. Although their limited biodistribution due the uptake by the liver and spleen, among their advantages it is important to highlight that are simple formulations with high biocompatibility and bioavailability. Lipidic systems can carry different payloads, for example, LNPs are widely used for the encapsulation of nucleic acids like miRNAs.

- Inorganic NPs

This kind of NPs can be formed by different materials as silica or gold, producing variations in their geometry, structure, and size. The used materials can provide them electromagnetic properties that are useful for photothermal therapy and diagnostic. Despite NPs have promising properties to cross the BBB, they can be optimised by their functionalization. Some examples are ligands like antibodies, aptamers, proteins and peptides or receptors.

**Fig. 3:**
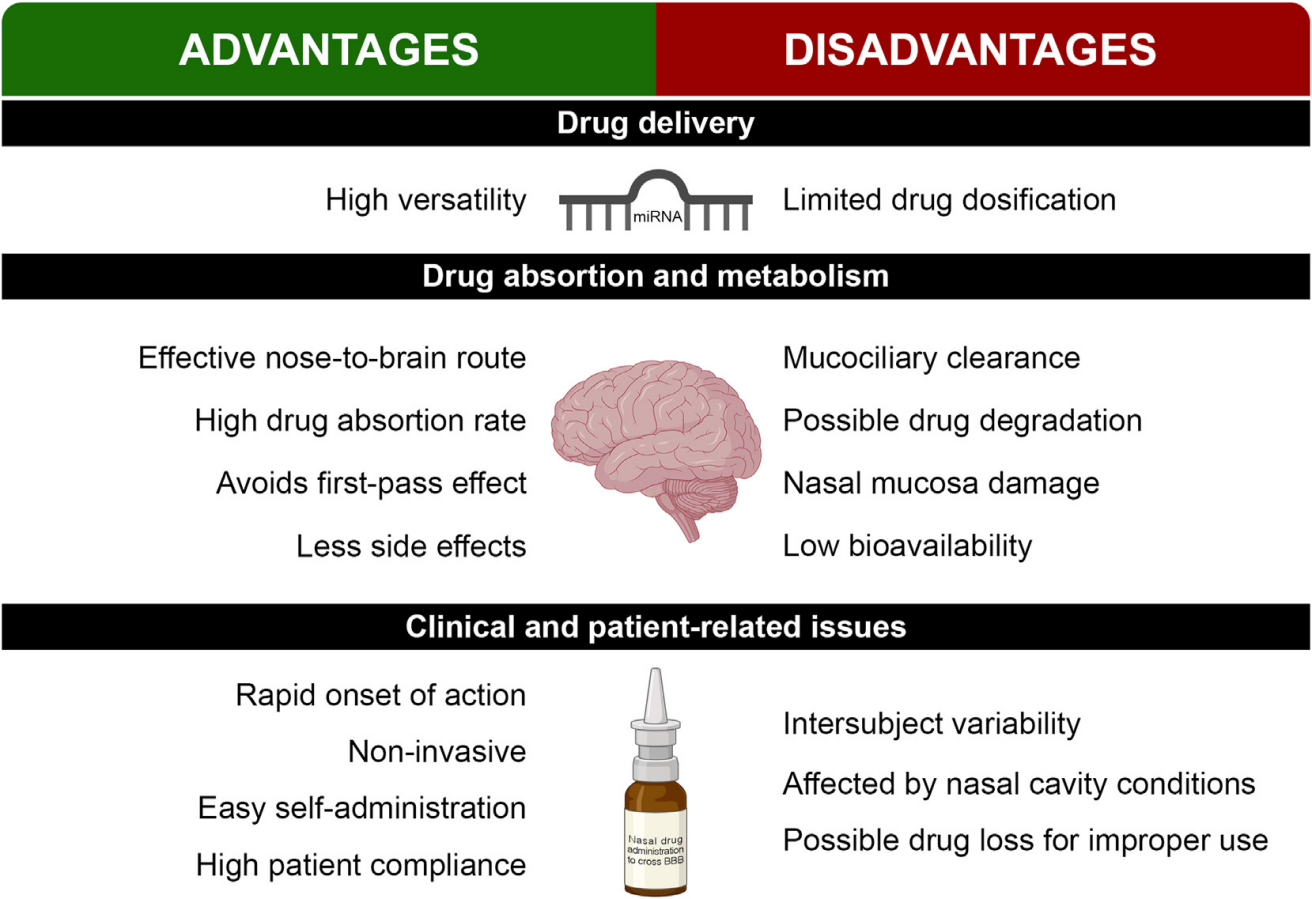
Advantages and disadvantages in nasal drug delivery.

There are several administration routes that can deliver drugs and molecules through the BBB (Diener et al., 2022). One of the most promising routes is the nasal, due to its unique characteristics (Fig. 3). As seen in Fig. 3, nasal administration provides a direct and effective nose-to-brain delivery of a wide range of drugs, small molecules, proteins or even miRNAs. However, it is not possible deliver high drug doses efficiently (Xie et al., 2019; Tai et al., 2022). This route has a high absorption rate due to the large mucosal surface area, without experimenting first-pass effect. Additionally, the action of the delivered molecules is produced with less side effects because of a minor drug accumulation in no targeting-tissues (Tai et al., 2022). The main problems the formulation can face once administered are the mucociliary clearance and the possible enzymatic drug degradation. The mucociliary clearance removes the drug from its absorption site, so developing a mucoadhesive formulation would be a proper solution. To avoid the enzymatic drug degradation, the nanocarrier should be prepared with enzyme inhibitors or protective shells (Tai et al., 2022).

Overall, nasal administration accomplishes a rapid onset of action with high patient compliance due to its non-invasiveness and easy self-administration; but its efficacy could also be reduced depending on the proper use of the device or the nasal cavity conditions of the patients (Tai et al., 2022).

## 4 Conclusion

As key regulators of cell behaviour in physiologic and pathological conditions, epigenetic modulation holds an immense potential for clinical application, in particular the use of miRNA as a pharmacological tool. Although naked miRNAs are susceptible to rapid systemic degradation, encapsulation of miRNAs into NPs can overcome these delivery challenges. Therefore, an enhanced targeting efficacy and reduced adverse effects are the main results, with a more significant effect if the target cell is difficult to access pharmacologically as the BBB. However, comparative studies only investigated the physicochemical properties of the NPs or their uptake by cells and it remains to be validated if these findings are also relevant in clinical medicine. Without a doubt, the creation of new therapeutic strategies focused on the brain parenchyma is needed since most of current therapies fail to pass efficiently the BBB.

## Declaration of competing interest

The authors declare the following financial interests/personal relationships which may be considered as potential competing interests:Alejandro Gonzalez-Candia reports article publishing charges was provided by University of O'Higgins. Alejandro Gonzalez-Candia reports a relationship with University of O'Higgins that includes: employment.
